# Smallpox Research Activities: U.S. Interagency Collaboration, 2001

**DOI:** 10.3201/eid0807.020032

**Published:** 2002-07

**Authors:** James W. LeDuc, Inger Damon, James M. Meegan, David A. Relman, John Huggins, Peter B. Jahrling

**Affiliations:** * Centers for Disease Control and Prevention, Atlanta, Georgia, USA; † National Institutes of Health, Bethesda, Maryland, USA; ‡ Stanford University, Stanford, California, USA; § U.S. Army Medical Research Institute of Infectious Diseases, Fort Detrick, Frederick, Maryland, USA

**Keywords:** smallpox, variola virus, vaccine, bioterrorism, vaccinia, orthopoxviruses

For the past 2 years, a team of investigators from the Centers for Disease Control and Prevention (CDC) has collaborated with scientists from the National Institutes of Health (NIH), the Food and Drug Administration, the Department of Defense (DOD), academic centers, and international partners to undertake a research agenda on variola virus, the etiologic agent of smallpox. Objectives of the program derive from a 1999 Institute of Medicine report that addressed the scientific needs for live variola virus (1). Progress in addressing these objectives has been peer reviewed annually by both a select committee organized by CDC and the World Health Organization (WHO) Advisory Committee on Variola Virus Research (2,3). A summary of accomplishments from the first year’s efforts was published in 2001 (4).

The events of September 11, 2001, coupled with the use of *Bacillus anthracis* as a bioterrorist weapon of mass destruction, have substantially increased concerns that variola virus may be similarly used and have added a sense of urgency to production of a new smallpox vaccine and to carrying out the smallpox research agenda. This report provides an update on progress during 2001.

## Vaccine

CDC has worked closely with the manufacturer of Dryvax (Wyeth, Marietta, PA), the 20-year-old smallpox vaccine held in stockpile, to ready all 15.4 million doses of vaccine for immediate distribution. Stocks of this vaccine were retested for potency by the manufacturer and with very few exceptions were found to remain fully potent. However, problems were detected with the diluent used to rehydrate the freeze-dried vaccine, and consequently all diluent has been replaced. A recent NIH study found that Dryvax vaccine could be diluted 1:5 and 1:10 and remain fully potent when administered to vaccinia-naïve persons (5). Either a 1:5 or 1:10 dilution of vaccine can be made in the original vaccine container, without the necessity of transfer to larger vials. Emergency vaccination plans now call for the vaccine to be used at a 1:5 dilution; thus, the existing stockpile could protect approximately 75 million persons. Sufficient additional diluent and needles are being purchased to support administration of the 75 million doses of vaccine.

To ensure that sufficient vaccine becomes available to protect the entire U.S. population, a contract established in 2000 by CDC with Acambis (Cambridge, MA) to produce and maintain a stockpile of 40 million doses of a new, MRC-5 (a diploid human lung cell line suitable for the production of viral vaccines) cell-culture–grown vaccinia vaccine was modified to reflect the need for expanded and accelerated production and human testing. The revised goal is now to produce >50 million doses by the end of 2002, with increased surge capacity for production of >180 million doses annually from 2003 on. Pilot lot production of the new vaccine is now under way, as are Phase 1 clinical trials. Phase 2 and 3 clinical trials are scheduled to begin later in 2002. Acambis expects to license the new vaccine by the end of 2003; however, it will be available for emergency use as an Investigational New Drug (IND) product as soon as it is manufactured. A second contract has been awarded to Acambis, in partnership with Baxter (Vienna, Austria), for production of 155 million doses of Vero cell-culture–produced vaccinia vaccine, for delivery by the end of 2002. The expanded use of the Dryvax vaccine, coupled with production of new vaccine from these two contracts, should result in sufficient vaccine for the entire U.S. population by the end of 2002.

Vaccinia vaccine is known to produce adverse events in a small number of recipients (6). In the spring of 2001, interested parties met to discuss management of vaccinia vaccine adverse events. Among the findings was the need to produce substantially larger stocks of vaccinia immune globulin, and suppliers are now being sought to produce approximately 30,000 adult treatment doses.

## Antiviral Drugs

An important recommendation from the Institute of Medicine report (1) was to identify antiviral drugs with activity against variola and related orthopoxviruses. The research team has been working toward the goal of identifying two distinct antiviral drugs with different mechanisms of action and effectiveness in the treatment of smallpox infection. One candidate, cidofovir, has been identified and its activity against variola virus demonstrated in in vitro assays. During 2001, an IND was filed for use of cidofovir in both the treatment of acute smallpox infection and the management of adverse events associated with vaccinia immunization. The team examined selected analogs of cidofovir and found some to be 25- to 150-fold more active than the parent compound in in vitro assays. These promising candidates will be included in future assays with animal models. More than 700 additional candidate antiviral drug compounds have been screened by in vitro assays for activity to various orthopoxviruses. Of these, >20 were found to hold promise against vaccinia and variola viruses and will be tested in animal models. In addition, Eli Lilly and Company (Indianapolis, IN) has provided access to agents from their portfolio of compounds for screening against variola virus. Access to such compounds, which are already being used in humans and routinely produced, could dramatically accelerate efforts to identify antiviral compounds that would be useful against vaccinia and variola viruses.

## Diagnostic Tests

The team continues to develop and validate assays to rapidly and accurately diagnose smallpox infection and the presence of variola virus, including protein-based tests to identify variola virus antigen and antivariola immunoglobulins, as well as specific nucleic acid detection tests. However, validation of prototype assays is hampered by the lack of access to relevant human clinical samples. DOD and international collaborating scientists worked successfully to identify a panel of monoclonal and polyclonal antibodies for detection and diagnosis of variola virus. An assay was developed for rapid and specific identification of variola virus with polymerase chain reaction (PCR) probes targeted to variola gene sequences. When these probes were used in the Smart Cycler (Cepheid, Sunnyvale, CA)/ TaqMan (Roche Molecular Systems, Inc., Indianapolis, IN) format, the limit of detection was validated by using the Bangladesh 1975 variola isolate and found to be approximately 483 copies. The assay was evaluated in a blinded study with 164 samples that included genomic DNA from 40 isolates of variola and 8 isolates of other orthopoxviruses at concentrations ranging from 0.1 to 10,000 pg/μL. No false-positive reactions were detected with any of the 37 nonvariola samples (100% specificity). Of the 127 samples containing variola DNA, 10 were considered negative, all at concentrations of <10 pg. The overall sensitivity was 92% at a limit of detection of 0.1 pg and 95% at a limit of detection of 1.0 pg. Preliminary data indicate that the assay is compatible with the ABI 7700 (Perkin-Elmer, Applied Biosystems Division, Foster City, CA) and the Light Cycler (Roche Diagnostic Corp., Indianapolis, IN) platforms, as well as colorimetric and electrochemical PCR enzyme-linked immunosorbent assay platforms. Collaborative work is continuing to evaluate and validate the assay to these various platforms.

## Genetic Diversity of Variola Virus

The need for additional sequence analyses of variola virus isolates was recognized both by the Institute of Medicine expert review (1) and the WHO Advisory Committee on Variola Virus Research Expert Committee on Orthopoxviruses. The WHO committee specifically recommended that, at a minimum, isolates from Congo and Somalia be fully sequenced before final destruction of variola virus. The team has developed a standardized method to extend PCR-restriction fragment-length polymorphism (RFLP) for the detection and differentiation of variola virus strains. From the WHO collection held at CDC, 45 variola isolates were selected on the basis of diverse geographic distribution and year of isolation. Twenty consensus primer pairs were used to produce 20 overlapping amplicons, which include 99.9% of the variola genome. The amplicons were digested with restriction endonuclease and resolved on acrylamide gradient gels, and the resulting RFLP patterns of the amplicons from the 45 isolates were compared. A composite dendrogram of all amplicon RFLP profiles differentiates variola major from variola minor, and the subclades within variola virus were generally clustered according to their geographic location or epidemiologic history. Unique RFLP patterns are generated on different amplicons of the variola isolates; these patterns can be used to differentiate variola strains and may be highly informative in epidemiologic studies. A database being built may also help in rapid recognition of variola or other orthopoxviruses that have been genetically engineered by the insertion of foreign genes.

On the basis of the WHO expert committee recommendations and the degree of diversity identified through the RFLP analyses, the team determined complete genomic sequences for eight isolates of variola virus from the WHO collection (named by geographic area and year of collection: Congo 70, Somalia 77, Nepal 73, India 64, Sumatra 70, Afghan 70, Horn 70, and Bangladesh 75). In addition to determining the full-length sequence of the most diverse isolates of variola virus, sequencing has been completed for >30 genes encoding various proteins from the other 37 isolates examined from the collection. All results and analyses are being integrated into a poxvirus-specific relational database suitable for public access (www.poxvirus.org). Finally, a new proteomics facility is being established at CDC to facilitate further characterization of variola virus isolates with the goal of providing essential information to assist in the rational design of antiviral drugs and therapeutic interventions. This facility is scheduled to be fully functional during 2002.

## Animal Model for Smallpox

Clinical manifestations of human smallpox are well described (7). However, attempts to establish an animal model that faithfully replicates human smallpox have historically been unsuccessful. In 2002, the interagency team began evaluation of nonhuman primate models to facilitate in vivo evaluation of antiviral drugs and other therapeutic interventions. A valid animal model will generate important clinical material to assist in validating smallpox diagnostic tests and will allow detailed investigation of the pathogenesis of smallpox infection. Earlier efforts had succeeded in infecting cynomolgus macaques with variola virus but failed to produce clinical illness fully consistent with human smallpox. Exposures of additional monkeys through a combination of both intravenous injection and aerosol exposure to high-titered (10^9^ PFU) variola virus succeeded in inducing infection, which led to fatal disease after a clinical course like that of smallpox in humans. Subsequent infections by the intravenous route alone led to fatal infection at only the highest infecting dose (10^9^ PFU), although monkeys receiving lower doses (10^6^ to 10^9^ PFU) did show evidence of variola infection, and virus dose was correlated with severity of disease course (7). Distribution of viral antigens by immunohistochemistry was correlated with replicating viral particles, observed by electron microscopy, and pathologic lesions resembling human smallpox. Analysis of daily specimens allowed detection of viral genomes in peripheral blood leukocytes and throat fluids by TaqMan PCR within 48 hours of exposure, suggesting the possibility of definitive diagnosis of smallpox during the prodrome. Overall, these results suggest that a valid animal model for smallpox may be feasible.

Collaborative studies with scientists from Stanford University allowed application of high-density DNA microarrays to measure and classify gene expression and to study the behavior of many genes simultaneously in monkeys experimentally infected with variola virus. Peripheral blood mononuclear cells were obtained from monkeys on the day of infection and multiple subsequent days. Gene expression analysis identified dramatic, highly choreographed response patterns and revealed several biological themes that appeared to be related to the outcome of infection (K. Rubins et al., Stanford University, unpub. data). Additional experimental infections of nonhuman primates with variola and other orthopoxviruses will be conducted. Potential benefits of host genome-wide expression profiling include early detection of infected persons, recognition of prognostic markers, rational development of novel therapeutic and prophylactic strategies, and determination of early signatures of a protective immune response to vaccination.

The team has continued investigating whether human chemokine receptors are involved as host factors in orthopoxvirus infections. Orthopoxviruses have no known unique viral attachment protein or cell surface receptor. Chemokine receptors, which are on the surface of leukocytes, are involved in directing leukocytes into areas of inflammation; other pathogens are known to use these receptors for cell entry. To determine the potential role of chemokines and their receptors in the pathogenesis of variola infection, experiments were performed by using a cell line transfected with genes encoding human CD4 receptors and subsequently transfected with human chemokine receptors. In low-multiplicity infection, the growth rate of variola was enhanced when human chemokine receptors were expressed. These and other observations suggest a possible role for chemokine receptors in the net growth and spread of variola virus. Investigations of the role of chemokines in the pathogenesis of variola infection will continue.

## Conclusion

World Health Assembly resolution WHA52.10 called for the destruction of all remaining stocks of variola virus by the end of 2002; however, a recent WHO review of research progress concluded that the live virus would be required beyond that date (8,9), a position supported by the 55th World Health Assembly. Specific scientific priorities that remain to be addressed include 1) obtaining further sequence data from the terminal regions of additional variola isolates, 2) continuing efforts to effectively detect variola virus infection and validate these procedures, 3) developing new drugs for smallpox treatment, 4) developing less reactogenic vaccines to protect against smallpox infection, and 5) validating an animal model of human smallpox to allow assessment of candidate drugs and vaccines for both efficacy and regulatory purposes.
