# Correction, Vol. 8, No. 7

**DOI:** 10.3201/eid0811.C10811

**Published:** 2002-11

**Keywords:** errata, erratum, correction

In Smallpox Research Activities: U.S. Interagency Collaboration, 2001 by James LeDuc et al., an error occurred in the text on Page 744, left column, under Diagnostic Tests, line 25. The information provided between lines 10 and 25 should be attributed to S. Ibrahim, U.S. Medical Research Institute of Infectious Diseases as unpublished data.

The corrected article appears online at http://wwwnc.cdc.gov/eid/article/8/7/02-0032_article.htm

We regret the omission.

